# Influence of Blood Collection Systems on Coagulation Tests

**DOI:** 10.5505/tjh.2012.59254

**Published:** 2012-12-05

**Authors:** Soner Yavaş, Selime Ayaz, S.Kenan Köse, Fatma Ulus, A.Tulga Ulus

**Affiliations:** 1 Türkiye Yüksek İhtisas Education and Research Hospital, Cardiovascular Surgery Clinic, Ankara, Turkey; 2 Türkiye Yüksek İhtisas Education and Research Hospital, Hematology Laboratory, Ankara, Turkey; 3 Ankara University Faculty of Medicine, Department of Biostatistics, Ankara, Turkey; 4 Atatürk Chest Disease and Thoracic Surgery Education and Research Hospital, Anesthesiology and Reanimation Clinic, Ankara, Turkey

**Keywords:** Coagulation testing, Glass tubes, Plastic tubes

## Abstract

**Objective:** Coagulation tests are influenced by pre-analytic conditions such as blood collection systems. Change of glass collection tubes with plastic ones will cause alteration of the test results.

The aim of this study was to compare three plastic blood collection tubes with a standard glass blood collection tube and each plastic collection tube with the other two for possible additional tube-to- tube differences.

**Material and Methods:** A total of 284 blood samples were obtained from 42 patients receiving warfarin during their routine controls, besides 29 healthy volunteers. Subgroup analyses were done according to health status.

**Results:** Our study demonstrated that different blood collection tubes have a statistically significant influence on coagulation tests. The magnitude of the effect depends on the tube used. However most of the tests performed on samples obtained from any tube correlated significantly with results obtained from other tube samples.

**Conclusion:** Although blood collection tubes with different brands or properties will have distinct effects on coagulation tests, the influence of these blood collection tubes may be relatively small to interfere with decision-making on dose prescription, therefore lack clinical importance. Correlations between the results showed that, one of these plastic blood collection tubes tested in our study, can be used interchangably for a wide variety of coagulation assays.

**Conflict of interest:**None declared.

## Abstract (tr)

**Amaç:** Koagülasyon testleri, farklı kan alma sistemleri gibi preanalitik durumlardan etkilenirler. Kullanılan cam tüplerin, plastik olanlarla değiştirilmesi, test sonuçlarında farklılık yaratabilir. Çalışmanın amacı 3 farklı plastik kan alma tüpünün, standart cam kan alma tüpü ile karşılaştırılmasıdır. Tüpler arası olası ek bir farklılığın saptanması açısından, plastik tüpler de ayrıca kendi aralarında karşılaştırıldı.

**Gereç ve Yöntemler:** Rutin kontrolleri sırasında varfarin tedavisi alan 42 hastadan ve 29 sağlıklı gönüllüden toplam 284 kan örneği alındı. Subgrup analizleri deneklerin sağlık durumlarına göre yapıldı.

**Bulgular:** Her ne kadar farklı marka veya özellikteki kan alma tüpleri koagülasyon testleri üzerinde belirgin etki yaratabilse de, bu etki doz ayarlamasını etkilemeyecek ve klinik önem arz etmeyecek kadar küçük olabilir. Sonuçlar arasındaki korelasyon, çalışmamızda kullanılan plastik kan alma tüplerinden herhangi birinin, bir çok koagülasyon testi için, diğerlerinin yerine kullanılabileceğini göstermektedir.

**Sonuç:** Çalışmamız farklı kan alma tüplerinin koagülasyon testleri üzerinde istatistiksel olarak anlamlı etkisi olduğunu gösterdi. Bu etkinin derecesi kullanılan tüpe göre değişmektedir. Fakat, herhangi bir tüpten alınan örneklerle gerçekleştirilen testlerin çoğunun, diğer tüplerden alınan örnekler ile belirgin korelasyon gösterdiği gözlendi.

## INTRODUCTION

Siliconized glass collection tubes have traditionally been used in the coagulation tests for the determination of International Normalized Ratio (INR), prothrombin time (PT) and coagulation factor levels.[1,2,3,4,5] However, the potential risk of sharp injury and biohazardous exposure due to broken glass during handling or centrifugation, rendered the need of newer plastic collection tubes and clinical laboratories gradually replaced glass collection tubes with plastic ones.[1,2,3,4] 

Since coagulation tests are influenced by pre-analytic conditions such as the blood collection systems, change of glass collection tubes with plastic ones raised concerns about the potential for in vitro activation of the clotting cascade, hence alteration of test results.[1,2,3,4,5,6,7,8,9,10,11,12,13] 

Therefore, we planned to compare three different plastic blood collection tubes with a standard glass blood collection tube which is used worldwide as in our hospital, to find out whether glass and plastic blood collection tubes have significantly different influence on coagulation tests, both in patients under oral anticoagulant therapy (OAT) and in healthy volunteers. Each plastic collection tube was also compared with each other for possible additional tube-to-tube differences. To the best of our knowledge, no study in current literature has also compared the plastic collection tubes at the same time.

## PATIENTS AND METHODS

A total of 284 blood samples were obtained from 42 patients receiving warfarin during their routine controls, as well as 29 healthy volunteers, upon approval from Education, Planning and Coordination Committee (Ethical Committee) in our hospital. Our study was performed according to the principles outlined in the appropriate version of 1964 Declaration of Helsinki and informed consent was obtained from each subject. 

Blood samples were taken using a 21G needle and BD Vacutainer reuseable standard size tube holder (Becton Dickinson, USA), allowing natural vacuum of tube to withdraw specimen into tube by clean venopuncture after 8-12 hours of fasting and prior to the daily dose of warfarin in the patient group. The test tubes contained sodium citrate 3.2%, with a ratio of one part anticoagulant to nine parts whole blood. The precedure was completed when vacuum no longer continued to withdraw. All samples were obtained from peripheral arm veins. For each case, 1 tube of blood sample was collected into a glass collection tube and 3 additional samples were collected into 3 different plastic collection tubes, in a random order. Tubes were delivered in the laboratory, checked for adequate tube filling, and centrifuged for 10 minutes at 2,000 G to prepare plateletpoor plasma (<10,000 platelets per microliter). In keeping accordance with the Clinical and Laboratory Standards Institute (formerly the National Committee for Clinical Laboratory Standards) guidelines, collection tubes were kept unopened at 18°C to 24°C before separation of cells from plasma. Hemolytic and/or lipemic samples were excluded. All samples were tested as fresh plasma and processed within 2 hours of collection to avoid the loss of activity of coagulation factors. All four samples from the same person were processed and analyzed at the same time. 

Patient demographics, including sex, age, primary diseases, and medication history were recorded. 

**Blood collection tubes were grouped as;**

*Tube Group I:* BD Vacutainer® Citrate Tubes - Glass (BD Vacutainer®, 9NC Sodium Citrate, 3.2%, 4.5 mL, Becton, Dickinson and Company, UK.), 

*Tube Group II:* VACUETTE® Blood Collection Tubes (Vacuette® Coagulation Tubes, 9NC Sodium citrate, 3.2%, 4 mL, Greiner Bio-One GmbH, Germany.), 

*Tube Group III:* BD Vacutainer® Plus Plastic Citrate Tube (BD Vacutainer® 9NC Sodium Citrate, 3.2%, 2.7 mL, Becton, Dickinson and Company, UK.), 

*Tube Group IV:* BD Vacutainer® Plus Plastic Citrate Tube (BD Vacutainer® 9NC Sodium Citrate, 3.2%, 1.8 mL, Becton, Dickinson and Company, UK.). Subgroup analyses were done according to health status as demonstrated below: 

*Group P:* Patients receiving warfarin therapy (n= 42, 59.2%), 

**Group H:** Healthy Volunteers (n= 29, 40.8%). 

Mean INR, PT%, PT, activated partial thromboplastin time (APTT), activated partial thromboplastin time ratio (APTT R) and fibrinogen levels were obtained both in healthy volunteers (Group H) and patients under OAT (Group P). 

D-Dimer, protein C, protein S, antithrombin, thrombin time, Factor V, Factor VII, Factor VIII, Factor IX, Factor X, Factor XI, Factor XII, plasminogen, Alpha 2 antiplasmin, activated protein C resistance test (APCR), Lupus antibodies (Lupus Ab) and von Willebrand factor antigen (vWF Ag) were available only in healthy volunteers.

## STATISTICAL ANALYSIS

utilized to determine the correlations between groups. Probability (p) values below 0.05 were considered significant. Confidence intervals (CI) were calculated at the 95% level. 

Correspondences of results between Tube Groups were undertaken using the Bland-Altman procedure, and Bland- Altman plots were performed to assess the magnitude of disagreement between the results, plotting the mean of the results for the two methods on the x axis against the arithmetic or percentage difference on the y axis [14].

## RESULTS

A total of 284 samples were obtained from 71 cases (29 men (40.8%), 42 women (59.2%)). Mean age of the patients was 44.4±14.6 years. 

**Without any subgrouping;**

When all mean values were compared, INR, PT, APTT and fibrinogen results obtained in Tube Group II were different but not significantly from the other groups (Table 1). 

**Figure 1:**
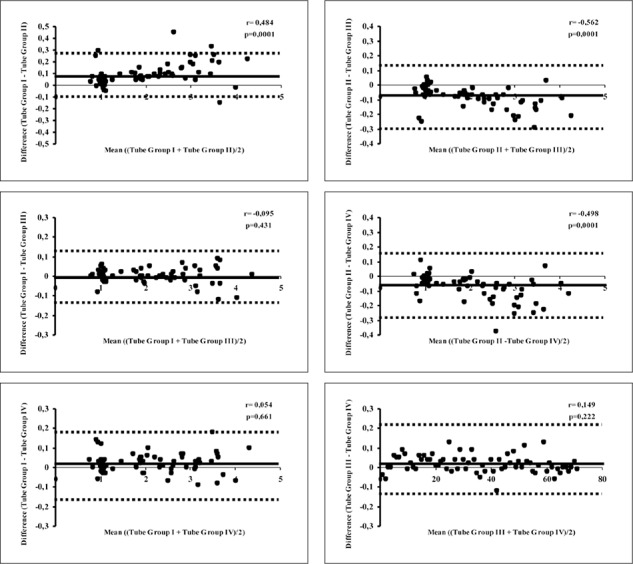
Correspondences between mean INR values according to Tube Groups without any subgrouping (Bland-Altman plots). Upper-Left: Tube Group I vs Tube Group II, Upper-Middle: Tube Group I vs Tube Group III, Upper-Right: Tube Group I vs Tube Group IV, Lower-Left: Tube Group II vs Tube Group III, Lower-Middle: Tube Group II vs Tube Group IV, Lower-Right: Tube Group III vs Tube Group IV. p<0.05: No correspondence.

Pearson correlation test showed significant correlations between total mean values. Using Bland-Altman procedure, significant correspondences were also valid between Tube Group I, III and IV. (Table 2) Correspondences between mean INR values were shown in **Figure 1** as Bland- Altman plots. 

When mean D-Dimer, protein C, protein S, antithrombin, thrombin time, Factor V, Factor VII, Factor VIII, Factor IX, Factor X, Factor XI, Factor XII, plasminogen, Alpha 2 antiplasmin, activated protein C resistance test (APCR), Lupus antibodies (Lupus Ab) and von Willebrand factor antigen (vWF Ag) values were compared, significant differences were observed between Tube Group II and the others, although some comparisons had significant correlations and correspondences (Table 1 and 2). 

**According to Group P and Group H;**

Demographic data of the patients were listed in Table 3. Since Group H consisted of young healthy volunteers, the only statistically significant difference was between mean ages (p= 0.0001). 

When mean values were compared, statistically significant differences existed especially between Tube Group II and the others (Table 4). When Pearson correlation test was performed, significant correlations were observed between mean values. But, Bland-Altman procedure showed poor correspondences between Tube Group II and the others (Table 5). 

Coagulation Factors were studied in Group H and variable results were obtained when mean values were com-pared (Table 1 and 2). While some of the tests were affected by plastic blood collection tubes, others were not.

## DISCUSSION

Different blood collection tubes will have an influence on laboratory tests and may result in changes in therapeutic dosage adjustment in patients receiving warfarin therapy. This may result in overdosing or underdosing which in turn may cause bleeding or thromboembolic complications.[15] 

Glass collection tubes are unblocked and have siliconized interior. On the other hand, plastic collection tubes have a double-wall technology (sandwich tubes – plastic within plastic) for reliable analysis results. The outer tube is made of polyethylene terephthalate and ensures a long shelf-life for the vacuum, while the inner tube is made out differof polypropylene and prevents the citrate solution from evaporating. Polypropylene is ideal for sensitive coagulation parameters, due to its inert characteristics.[1j] 

There are conflicting reports about the effects of plastic blood collection tubes on coagulation testing and most are limited to PT analyses.[1,5,12,16,17,18,19,20,21,22,23] Previous studies reported significant differences in thrombin time and PT test results.[1,5,12] 

The aim of our study was to determine whether conversion to plastic tubes from glass tubes would result in significant differences in laboratory results. Since no report has compared plastic collection tubes at the same time, we also focused on comparing each plastic collection tube with each other. 

Our study demonstrated that different blood collection tubes had a statistically significant influence on coagulation tests. The magnitude of the effect depended on the tube used. 

It has been suggested that INR differences below 10% do not seriously interfere with oral anticoagulant dosage regulation.[6,23] Our study showed that the mean INR values increased or decreased by the influence of differ of differ ent blood collection systems. Mean INR value was lowest in Tube Group II. Values obtained in Tube Group II and IV were 4.1% and 0.3% lower, respectively, and in Tube Group III was 0.02% higher than Tube Group I. Although statistical differences not existed in INR Group I, III and IV, values were nearly equal in practice. 

Although, some of the statistical analyses showed no differences for some of the assays like Alpha 2 antiplasmin, APCR, protein S or Lupus Ab between different tubes,poor correlations were observed. It is unclear why some of the tests would be affected by plastic blood collection methods while others would not. Type or International Sensitivity Index (ISI) of the reagent used, blood-tube surface interaction, the dynamic properties of the coagulation factors, use of individual vacuum tubes or clinically insignificant differences in tube blood volume (although all samples were checked for adequate filling) are some of the factors affecting the results.[1,2,5,7,8,10,11,12,24,25,26] 

Besides, as seen in Tube Group II, different brands may also have distinct effects on coagulation tests. 

Tube Group II was the only group with a different brand, but differences related to Tube Group II did not reach clinical significance because the difference below 10% does not seriously interfere with oral anticoagulant dosage regulation. [6,23] 

Correlations between the results showed that, plastic blood collection tubes can be used in place of glass tubes or instead of the other plastic tubes for a wide variety of coagulation assays but the clinicians should be aware of the fact. 

The influence of blood collection tubes on a single coagulation analyzer using a single thromboplastin reagent with a constant ISI, may be insufficiently small to interfere with decision-making on dose prescription. On the other hand it must be kept in mind that probably the combination of multiple systematic variables such as different brands, reagents or analyzers may cumulatively lead to important INR differences. To reduce the resultant total error from system combinations, the influence of the blood collection systems may have to be eliminated. Detection of inter-laboratory multicenter calibration standards for the establishment of an international reference will also be helpful. 

It should be a note of caution that, when any laboratory plans to change their blood collection method, clinicians must be alerted about the new method. Actually, the best way to gain experience with a new blood collection method may be the use of both the old and new tubes simultaneously for an adaptation period to avoid unmeant trouble. Since analytical or statistical significance is only numerical data, the most important judgment is clinical experience based on patient-dose-response triangle. 

Conflict of Interest Statement

None of the authors have any conflicts of interest, including specific financial interests, relationships, and/or affiliations, relevant to the subject matter or materials included.

## Figures and Tables

**Table 1:**
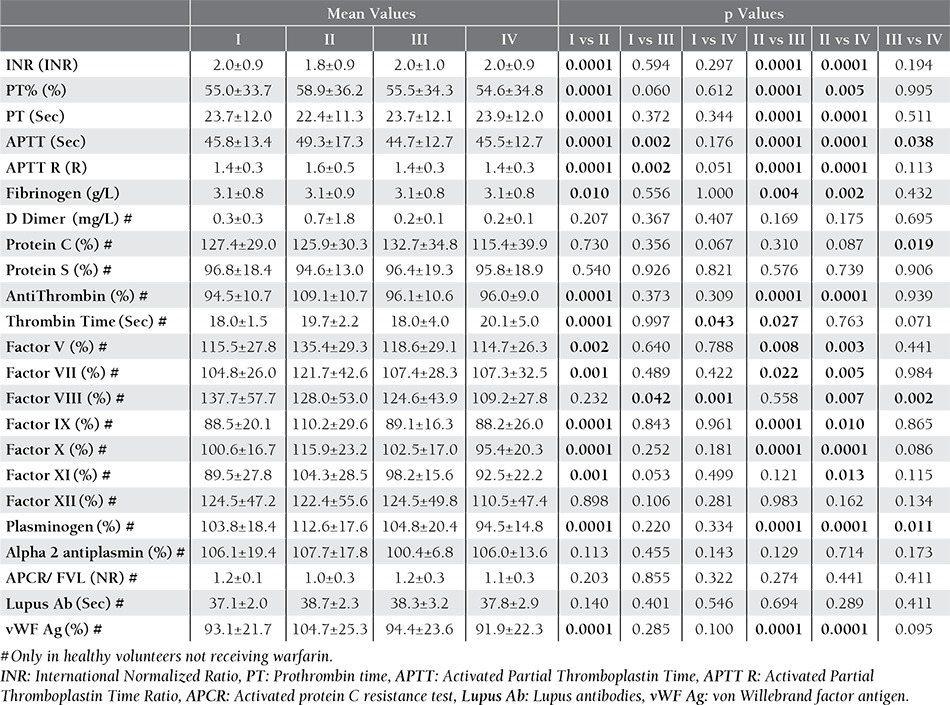
Comparison of tube groups without any subgrouping.

**Table 2:**
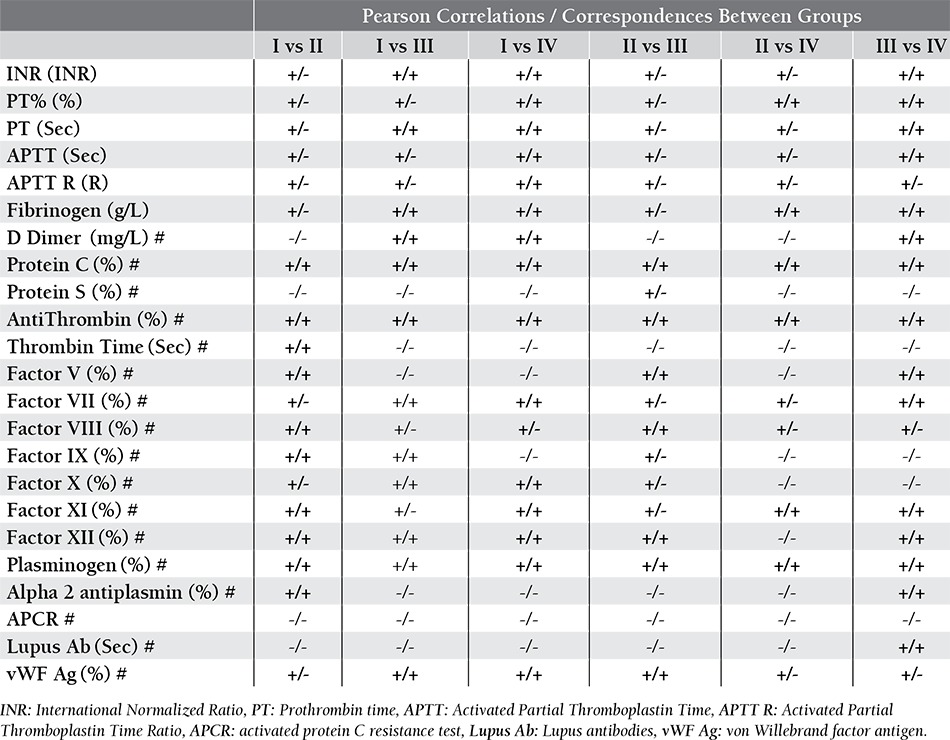
Correlations and correspondences between tube groups without any subgrouping (Correlations/Correspondences).

**Table 3:**
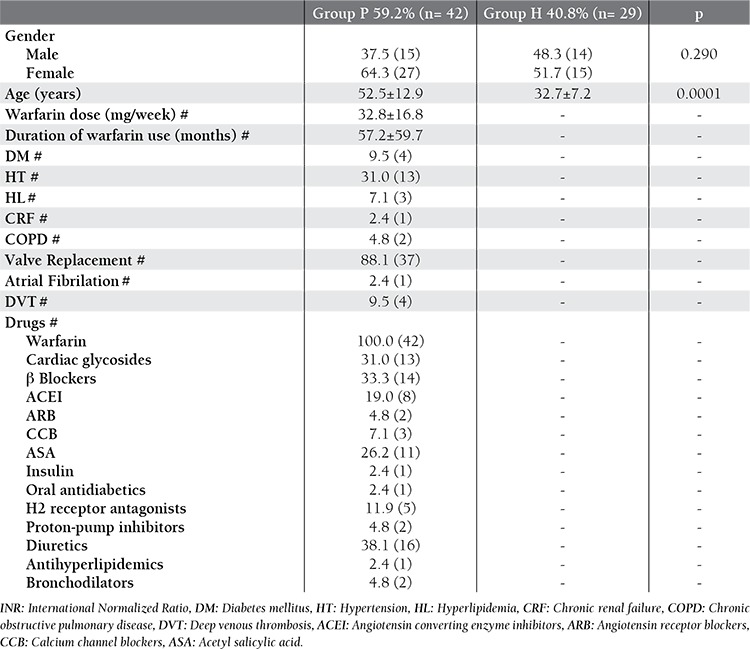
Demografic data according to Group P and H.

**Table 4:**
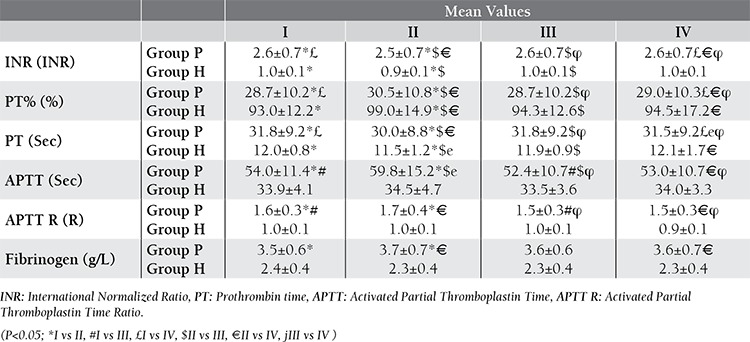
Comparison of tube groups according to Group P and H.

**Table 5:**
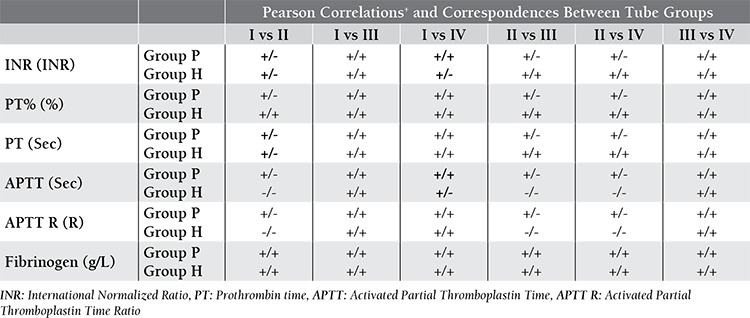
Correlations and Correspondences Between Tube Groups according to Group P and H (Correlations/Correspondences).
